# HDL functions and their interaction in patients with ST elevation myocardial infarction: a case control study

**DOI:** 10.1186/s12944-020-01260-4

**Published:** 2020-04-15

**Authors:** Himani Thakkar, Vinnyfred Vincent, Ambuj Roy, Sandeep Singh, Lakshmy Ramakrishnan, Mani Kalaivani, Archna Singh

**Affiliations:** 1 Department of Biochemistry, All India Institute of Medical Sciences, Room No. 3044, New Delhi, 110029 India; 2 Department of Cardiology, All India Institute of Medical Sciences, New Delhi, India; 3 Department of Cardiac Biochemistry, All India Institute of Medical Sciences, New Delhi, India; 4 Department of Biostatistics, All India Institute of Medical Sciences, New Delhi, India

**Keywords:** Cholesterol efflux capacity, Paraoxonase-1, Apolipoprotein A-I, High density lipoprotein, Atherosclerosis

## Abstract

**Background:**

Recent studies emphasize the importance of HDL function over HDL cholesterol measurement, as an important risk for cardiovascular diseases (CVD). We compared the HDL function of patients with acute coronary syndrome (ACS) and healthy controls.

**Methods:**

We measured cholesterol efflux capacity of HDL using THP-1 macrophages labelled with fluorescently tagged (BODIPY) cholesterol. PON1 activities toward paraoxon and phenyl acetate were assessed by spectrophotometric methods.

**Results:**

We recruited 150 ACS patients and 110 controls. The HDL function of all patients during acute phase and at six month follow-up was measured. The mean age of the patients and controls was 51.7 and 43.6 years respectively. The mean HDL cholesterol/apolipoprotein A-I levels (ratio) of patients during acute phase, follow-up and of controls were 40.2 mg/dl/ 112.5 mg/dl (ratio = 0.36), 38.3 mg/dl/ 127.2 mg/dl (ratio = 0.30) and 45.4 mg/dl/ 142.1 mg/dl (ratio = 0.32) respectively. The cholesterol efflux capacity (CEC) of HDL was positively correlated with apolipoprotein A-I levels during acute phase (r = 0.19, *p* = 0.019), follow-up (r = 0.26, *p* = 0.007) and of controls (r = 0.3, *p* = 0.0012) but not with HDL-C levels (acute phase: r = 0.07, *p* = 0.47; follow-up: r = 0.1, *p* = 0.2; control: r = 0.02, *p* = 0.82). Higher levels of cholesterol efflux capacity, PON1 activity and apolipoprotein A-I were associated with lower odds of development of ACS. We also observed that low CEC is associated with higher odds of having ACS if PON1 activity of HDL is also low and vice versa.

**Conclusion:**

ACS is associated with reduced HDL functions which improves at follow-up. The predicted probability of ACS depends upon individual HDL functions and the interactions between them.

## Introduction

High density lipoprotein-cholesterol (HDL-C) levels have been associated with atheroprotective effects in epidemiological studies [1]. However, result of multiple clinical trials with HDL-C raising agents (niacin and CETP inhibitors) have shown no cardiovascular benefit with increase in HDL levels [2, 3]. Studies using Mendelian randomisation have also refuted inverse association of HDL-C levels with cardiovascular diseases (CVD) [4, 5]. These findings, and the knowledge of the complex nature of HDL as a heterogenous group of molecules containing a large number of proteins with several pleiotropic functions, has brought the focus on assessing HDL function in addition to HDL-C levels for CVD risk stratification [6, 7].

Cholesterol efflux capacity of HDL allows it to remove excess cholesterol from the macrophages by the process of reverse cholesterol transport and promote its excretion from the body though bile. This property of HDL was found to be associated with lower CVD risk independent of the HDL-C concentration [8] HDL particles also have antioxidative properties and prevent oxidative modification of LDL particles that induce a proinflammatory profile in the vessel wall [9].

Our study aimed to analyze the cholesterol efflux capacity and antioxidative activity (as reflected in Paraoxonase 1(PON1) activity) of HDL in patients of acute coronary syndrome. The study objectives were to compare the HDL functions between healthy controls and patients presenting with acute coronary syndrome, evaluate the changes in the functional parameters after six months of treatment followed by an assessment of the relationship of both CEC and PON1 activity with HDL-cholesterol and Apo A-I. We have also studied the interaction between HDL functions that we analyzed, as predictors of the probability of development of acute coronary syndrome.

## Methods

### Study participants

We recruited 150 ACS cases and 110 control subjects. Patients presenting with ACS were recruited from the Cardiothoracic Centre at All India Institute of Medical Sciences, New Delhi, India. Acute Coronary Syndrome was defined as ischemic symptoms with ST segment elevation (more than 1 mm ST segment elevation in contiguous limb leads/more than 2 mm ST segment elevation in precordial leads) (STEMI) or with raised cardiac biomarkers (troponin) with or without ST segment changes in ECG (NSTEMI). Patients with inflammatory or autoimmune disorders, on thyroid medication or on statins were excluded. Our study included only patients who were diagnosed with STEMI. The control subjects included in the study were healthy adults with no clinical cardiovascular disease and not on any lipid-lowering therapy. Control group comprised of both volunteers with no history of cardiovascular diseases and subjects in whom no coronary occlusion was observed after undergoing CT angiography. Subjects with any autoimmune disease or chronic or acute infectious or inflammatory disorders were excluded from the study. The study protocol was approved by Institute Ethics Committee of All India Institute of Medical Sciences, New Delhi (Reference Number: IESC/T-380/17.10.2014). Signed informed consent was obtained prior to enrolling in the study. The participants did not receive any stipend for taking part in the study.

### Blood sample collection and biochemical parameters

Blood samples were collected at the time of presentation with ACS before administration of statins. The follow-up blood samples were collected at six months while on treatment, which included a statin (atorvastatin) along with other cardioprotective drugs as per treating physician prescription. Samples were centrifuged at 2500×g for 20 min, and after separation of serum, multiple aliquots of serum were immediately stored at minus eighty degree Celsius (− 80 °C) until further processing. Lipid profile of all participants were done using the AU480 auto analyzer (Beckman Coulter, California, United States). Apolipoprotein A-I (apo A-I), Apolipoprotein B (apo B) and high sensitive C reactive protein (hsCRP) levels were determined using immunoturbidimetric assay kits by Randox. The assay was performed in biochemical autoanalyzer according to the protocol described in the kit manual. Two levels of controls were assayed to confirm for accuracy and reproducibility. Sensitivity for the apo A-I, apo B and hsCRP kits were 5.78 mg/dl, 11.5 mg/dl and 0.27 mg/l. The intra assay CV for apo A-I, apoB and hsCRP assay was 3.08% (*n* = 20), 2.20% (n = 20) and 2.50% (n = 20). Inter assay CV for apo A-I, apoB and hsCRP assay was 3.10% (n = 20), 2.58% (n = 20) and 2.35% (n = 20).

### Determination of HDL functions

Cholesterol efflux capacity.

Macrophage specific cholesterol efflux was performed according to a previously described protocol with modifications [10]. Apo B depleted serum isolated using 20% polyethylene glycol was used as cholesterol acceptor in the assay. To measure the cholesterol efflux capacity of HDL, THP-1 human monocytes were differentiated into macrophages by the addition of 50 nM phorbol myristate acetate (PMA). Expression of ATP binding cassette transporter 1 (ABCA1) was analyzed using real-time PCR after differentiation. Differentiated THP-1 macrophages were labelled with BODIPY cholesterol. Labelling was done by incubating the cells in labelling media containing 0.025 mM BODIPY-cholesterol, 0.1 mM unlabeled cholesterol and 10 mM methyl beta cyclodextrin for 1 h followed by equilibration in Roswell Park Memorial Institute (RPMI) 1640 medium containing 0.2% bovine serum albumin for 15 h. Thereafter, cells were incubated with apo B depleted serum for 4 h. Thereafter, media was removed, centrifuged and fluorescence intensity was measured (excitation 482 nm, emission 515 nm). To calculate percent efflux, fluorescence intensity of the cells was also measured after equilibration time by solubilizing the cells in sodium deoxycholate. Cholesterol efflux capacity was calculated as [(fluorescence intensity with acceptor- fluorescence intensity without acceptor)/fluorescence intensity of cells after equilibration time].

Pooled serum from five healthy volunteers was used with every plate to correct for inter-assay variation, and values for cholesterol efflux from patient’s serum were normalized to this pooled serum values in subsequent analyses. All samples were run in triplicates. Inter assay coefficient of variation (CV%) was 12% (*n* = 27).

Paraoxonase 1 (PON1) activity.

Paraoxon-hydrolyzing activity of PON1 was assessed as the rate of formation of p-nitrophenol by hydrolysis of paraoxon. Activity was measured using 5 μL of freshly thawed serum from stored aliquots in 100 mM Tris- Cl buffer (pH 8) and 2 mM CaCl_2_ containing 4 mM paraoxon. Nonenzymatic hydrolysis of paraoxon was subtracted from the rate of hydrolysis by serum. Activity was calculated using a molar extinction coefficient (E412) of 18,290 M^− 1^ cm^− 1^ and expressed in U/mL. One unit of the paraoxonase activity equals 1 nmol of p-nitrophenol produced/min/mL.

Phenyl acetate was used as a substrate to study arylesterase activity of PON1. Rates of hydrolysis of phenyl acetate was determined spectrophotometrically at 270 nm using diluted serum. Reaction buffer included 8 mM phenyl acetate and 1 mM CaCl_2_ in 50 mM Tris HCl, pH 8.0. The amount of phenyl acetate hydrolyzed was calculated from the molar absorptivity, 1310 M^− 1^ cm^− 1^. One unit of arylesterase activity is defined as 1 μmol of phenyl acetate hydrolyzed per minute per mL. For estimation of paraoxon-hydrolyzing and phenyl acetate hydrolyzing activities of PON1, all samples were run in triplicates.

### Statistical analysis

Distribution pattern of the data for the variables was analyzed using Shapiro-Wilk test. Data with normal distribution were expressed as mean with standard deviation (SD) and nonparametric data were represented as median with interquartile values. For the categorical variables, results were presented in percentage. Student t-test and Mann-Whitney U test were performed to estimate the significance in the difference in HDL functional between two groups for parametric and non-parametric data respectively. Paired t-test was used to compare the HDL functionality parameters in ACS patients at two time points. Linear regression analysis was performed to test the association of cholesterol efflux capacity and antioxidative activity with apolipoprotein A-I. Correlation coefficient (r) and its corresponding *p* value were estimated using Pearson correlation coefficient separately and combined for cases (ACS) and controls.

Logistic regression was performed using control and ACS group as dependent variables and apolipoprotein A-I and HDL functions as independent variables. Odds ratio and their 95% confidence intervals (CI) were estimated per SD change in variable. Multivariate analysis was done where, in model 2, adjustment was done for cardiovascular risk factors like age, body mass index (BMI), gender, smoking, hypertension, diabetes and LDL-C. In model 3, additional adjustment for HDL -C level was done. Interaction between two continuous variables (i.e. HDL functions) was analyzed by running the logistic regression for interaction, *margins* and *marginsplot* commands were used for creating two-way contour to graph predictions from a model that includes an interaction between two continuous variables.

Two-sided test with *p* value < 0.05 was considered statistically significant. R Core Team (2018, R: A language and environment for statistical computing; R Foundation for Statistical Computing, Vienna, Austria; URL https://www.R-project.org/) software, Graph pad prism 6 (GraphPad software, California, USA) software, STATA 12.2/MP software and Adobe Illustrator (Adobe Systems, San Jose, CA, USA) software were used to perform the statistical analysis and creation of figures.

## Results

### Participants and biochemical parameters

The characteristics of the patients and healthy subjects and their biochemical parameters are summarized in the Tables 1 and 2 respectively. All ACS patients included in the study had ST segment elevation myocardial infarction (STEMI). There was a significant difference in the mean age of control group and ACS group (*p* < 0.001). There were more male subjects in ACS group than in control group. The ACS group had higher proportion of participants with diabetes and hypertension. They also had a higher percentage of smokers (*p* < 0.0001).

**Table 1:** Clinical characteristics of participants

Parameters	Control *n* = 110	ACS baseline *n* = 150	*p* value
**Age (years)**	43.6 ± 10.5	51.7 ± 11.4	< 0.001
**BMI (kg/m**^**2**^**)**	25.1 ± 3.6	25.3 ± 3.5	ns
**Gender** **Male/ Female n**	65/45	134/18	
**Smoking n (%)**	10 (9)	83 (55)	< 0.001
**Diabetics n (%)**	13 (11.8)	42 (27.8)	< 0.001
**Hypertensive n (%)**	20 (18.1)	45 (29.8)	0.03
**Medications**			
**Antihypertensive n (%)**	15 (13.6)	38 (25.3)	
**Anti-diabetic n (%)**	13 (11.8)	42 (27.8)	

Data expressed as mean ± standard deviation and total (percentage). *ACS* Acute coronary syndrome, *BMI* Body Mass Index

**Table 2:** Biochemical parameters of participants

Parameters	Control (*n* = 110)	ACS baseline (*n* = 150)	ACS follow-up (*n* = 100)
**Apo A-I (mg/ml)**	142.1 ± 43.3	112.5 ± 27.3***	127.2 ± 36.6^###^
**Apo B (mg/ml)**	72.3 ± 22.4	83.9 ± 28.9***	62.7 ± 20.5^###^
**Total cholesterol** **(mg/dl)**	161.2 ± 37.1	169.3 ± 42.0	137.0 ± 39.8^###^
**HDL-C (mg/dl)**	45.4 ± 9.6	40.2 ± 7.2***	38.3 ± 9.0
**LDL-C (mg/dl)**	104.0 ± 25.4	107.3 ± 34.9	80.8 ± 29.3^###^
**VLDL-C (mg/dl)**	16.9 ± 7.2	20.6 ± 11.2*	15.8 ± 11.1^###^
**TG (mg/dl)**	117.3 ± 45.2	126.8 ± 67.8	134.6 ± 90.2

Data are expressed as mean ± standard deviation. *ACS* Acute coronary syndrome, *Apo A-I* Apolipoprotein A-I, *Apo B* Apolipoprotein B, *HDL-C* HDL-cholesterol, *LDL-C* LDL- cholesterol, *VLDL-C* Very Low Density lipoprotein-cholesterol, *TG* Triglycerides. *represents significance value for comparison between control and ACS patients. ^#^represents significance value for comparison between ACS baseline and ACS follow-up patients. *p < 0.05; **p < 0.01; ***p < 0.001, ^#^*p* < 0.05; ^##^*p* < 0.01; ^###^*p* < 0.001

Patients presenting with ACS had significantly lower HDL-C (45.4 ± 9.6 vs 40.2 ± 9.6 mg/dl, *p* < 0.001) and apolipoprotein A-I (142.1 ± 43.3 vs 112.5 ± 27.3 mg/dl, *p* < 0.001) levels as compared to control subjects. ACS patients had significantly higher apo B levels as compared to controls. No significant difference was observed in total cholesterol (TC), LDL-C, very low density lipoprotein cholesterol (VLDL-C) and triglycerides (TG) between two groups overall.

### HDL functions

**Fig. 1:**
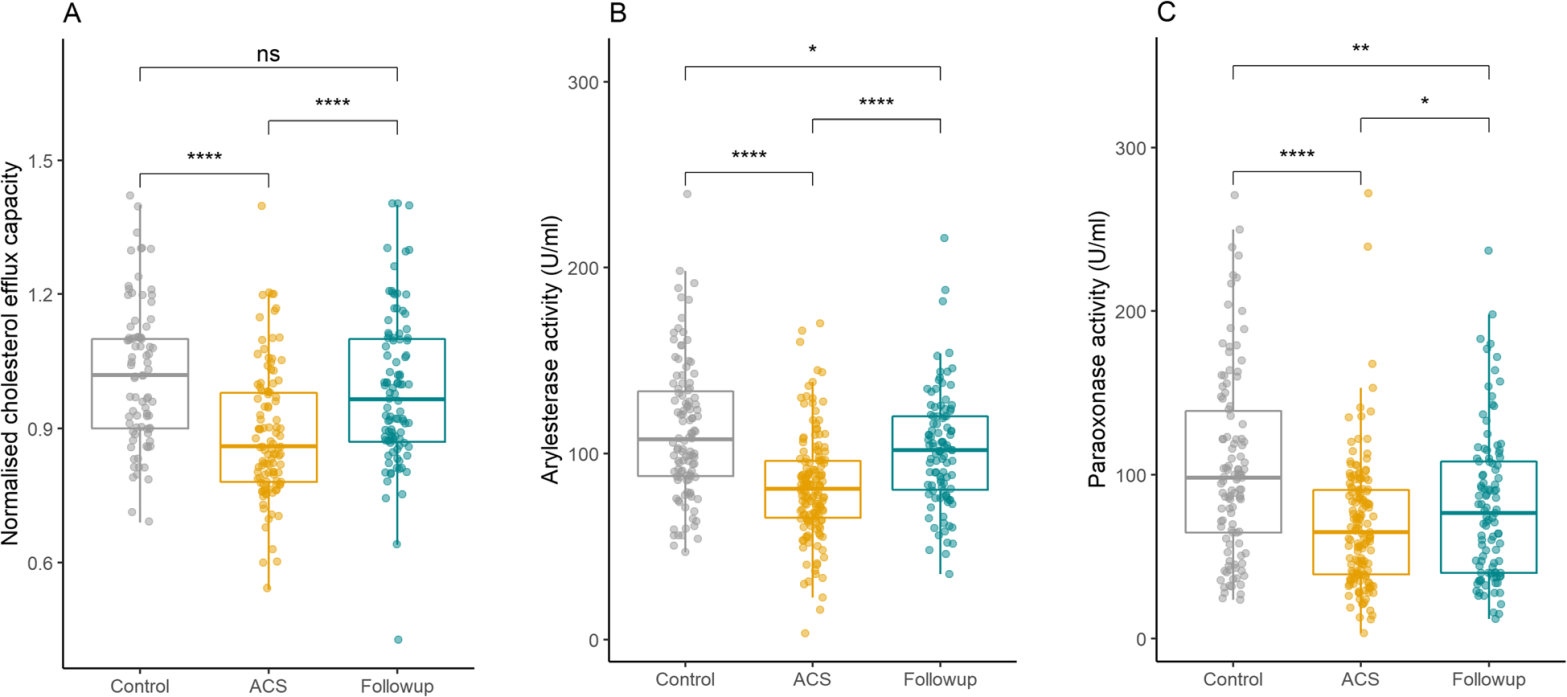
HDL functions in controls, ACS baseline and ACS follow-up. **a** HDL mediated cholesterol efflux capacity measured using BODIPY-cholesterol loaded THP-1 macrophages, **b** Arylesterase activity (phenyl acetate hydrolyzing activity) and **c** Paraoxonase activity (paraoxon-hydrolyzing activity) of PON1 in control and ACS patients at baseline and follow-up. Statistical analysis to determine the difference between two groups was performed using student t test. Data are shown as mean ± SD. Stars represents significance. *p < 0.05, **p < 0.01, ****p < 0.0001. ns represents non-significant

We observed that ACS patients had significantly lower HDL cholesterol efflux capacity, 0.88 ± 0.15 Arbitrary Units (AU) in ACS and 1.02 ± 0.16 AU in control subjects (*p* < 0.001) (Fig. 1a). Phenyl acetate hydrolyzing activity of PON1 was significantly lower in ACS patients (112.0 ± 36.3 vs 82.1 ± 27.9 U/mL, *p* < 0.001) (Fig. 1b). Paraoxon-hydrolyzing activity of PON1 was significantly reduced in ACS patients (105.1 ± 56.4 U/mL in controls and 69.1 ± 38.8 U/mL in ACS subjects) (Fig. 1c).

Smokers with ACS had significantly lower PON1 phenyl acetate hydrolyzing activity compared to non-smokers in ACS group (76.7 vs 88.7 U/mL, *p* = 0.008). CEC was lower in smokers (0.87 ± 0.15 AU) than in non-smokers (0.89 ± 0.14 AU) within the ACS group but the difference was not statistically significant. HDL functions were also found to be significantly lower in male and female ACS subjects compared to male and female controls respectively (Table 3).

**Table 3:** Comparison of HDL functions

HDL function	Control (*n* = 110)	ACS baseline (*n* = 150)	ACS follow-up (*n* = 100)
**Cholesterol efflux capacity (A.U.)**	1.02 ± 0.16	0.88 ± 0.15***	0.94 ± 0.17^###^
Male	1.04 ± 0.16	0.9 ± 0.14*	0.99 ± 0.19^###^
Female	1.0 ± 0.14	0.87 ± 0.15*	0.93 ± 0.19
**Paraoxonase activity (U/ml)**	105.1 ± 56.4	69.1 ± 38.8***	81.1 ± 45.5^###^
Male	107.1 ± 56.2	69.5 ± 40.0*	82.4 ± 46.5^###^
Female	101.9 ± 57.1	65.5 ± 29.1*	72.1 ± 37.5
**Arylesterase activity (U/ml)**	112.0 ± 36.3	82.1 ± 27.9***	101.8 ± 30.6^###^
Male	113.8 ± 38.8	81.0 ± 28.1*	100.4 ± 31.0^###^
Female	109.4 ± 32.7	90.2 ± 26.6*	111.8 ± 26.3^###^

Data are expressed as mean ± standard deviation. *ACS* Acute coronary syndrome, Paraoxonase activity represents paraoxon-hydrolyzing activity of PON1; Arylesterase activity represents phenyl acetate hydrolyzing activity of PON1. *represents significance value for comparison between control and ACS subjects. ^#^represents significance value for comparison between ACS baseline and ACS follow-up subjects. *p < 0.05; **p < 0.01; ***p < 0.001, ^#^*p* < 0.05; ^##^*p* < 0.01; ^###^*p* < 0.001

### HDL functions in ACS patients at follow-up

The follow-up samples from 100 patients were available. All ACS patients enrolled in the study were statin naïve and were on high dose statin after they were diagnosed with ACS. 45 were lost to follow-up and 5 had a second event of ACS or had died. A significant decrease in body mass index was observed after six months follow up (25.69 ± 3.9 vs 24.7 ± 4.0 kg/m^2^, *p <* 0.001). In the study, out of 100 ACS patients who were followed up, 62 were smokers at presentation and out of these 62, 37 quit smoking by the end of follow up.

Change in lipid profile after therapy is summarized in Table 2. A significant reduction was observed in total cholesterol, VLDL cholesterol and LDL-C levels after therapy. HDL-C also decreased after treatment, but the decrease was not significant; however, there was a significant increase in apolipoprotein A-I levels. Triglyceride levels also decreased after therapy, but the decrease was not significant. Apolipoprotein B levels were significantly reduced after six months. The patients at follow-up had significantly lower total cholesterol, apo B and LDL-C levels compared to control subjects. They also had significantly lower apo A-I levels and HDL-C levels compared to control subjects.

We observed a significant increase in cholesterol efflux capacity at follow up after six months in ACS patients (0.89 ± 0.14 vs 0.98 ± 0.17 AU; *p* < 0.001). The efflux capacity of ACS follow-up and control subjects were found to be similar (Fig. 1a). A significant improvement in PON1 phenyl acetate hydrolyzing (Baseline: 85.4 ± 30.2; Follow up: 101.8 ± 30.6 U/mL; *p* < 0.001) (Fig. 1b) and paraoxon-hydrolyzing activity (Baseline: 69.1 ± 42.; Follow-up 81.1 ± 45.5 U/mL; *p* < 0.001) (Fig. 1c) was observed after six months of therapy. Although there was marked increase in paraoxon-hydrolyzing and phenyl acetate hydrolyzing activity of PON1 in ACS patients after statin therapy, it was still significantly lower compared to control subjects.

### Correlation between HDL function and apolipoprotein A-I

**Fig. 2:**
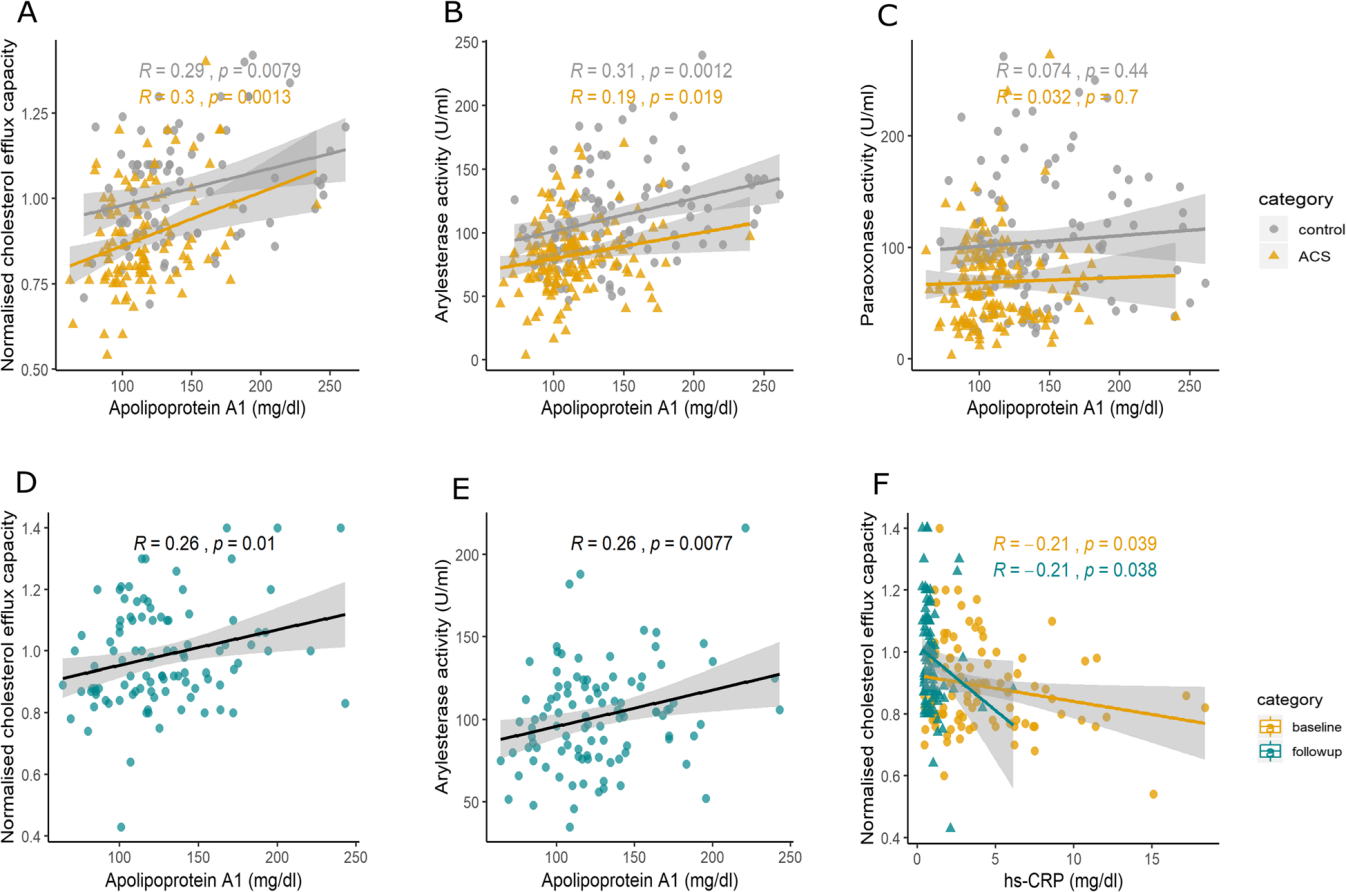
Correlation of HDL functions with apolipoprotein A-I**.** Correlation of (**a**) cholesterol efflux capacity and (**b**) Arylesterase activity (phenyl acetate hydrolyzing activity), (**c**) paraoxonase (paraoxon-hydrolyzing activity) activity of PON1 with apolipoprotein A-I in control, ACS subjects at baseline and (**d, e**) follow-up. Correlation of (**f**) cholesterol efflux capacity with hs-CRP levels in ACS patients at baseline and follow-up. Pearson correlation coefficient (R) and *p* value are shown. Line represents the regression line

Linear regression analyses showed a significant positive correlation between cholesterol efflux capacity and apolipoprotein A-I (r = 0.39. *p* = 0.001), and a similar correlation was observed in control (r = 0.29, *p* = 0.0079), ACS baseline (r = 0.3, *p* = 0.0013) (Fig. 2a) and ACS follow-up (r = 0.26, *p* = 0.01) groups separately (Fig. 2d). However, no correlation was observed between CEC and HDL-C levels in control (r = 0.02, *p* = 0.82) or ACS group (acute phase: r = 0.07, *p* = 0.47).

A strong positive correlation was observed between phenyl acetate hydrolyzing activity of PON1 and the levels of apolipoprotein A-I in controls (r = 0.3, *p* = 0.0012), ACS baseline (r = 0.19, *p* = 0.019) (Fig. 2b) and ACS follow-up groups (r = 0.26, *p* = 0.007). Figure 2e). We did not observe any correlation between paraoxon- hydrolyzing activity of PON1 and apolipoprotein A-I levels and HDL- C levels (Fig. 2c).

hs-CRP levels were measured as an index of inflammation in acute coronary syndrome subjects. Cholesterol efflux capacity showed an inverse correlation with hs-CRP levels at follow-up (r = − 0.2, *p* = 0.03) and the same trend was also observed in the baseline ACS data (r = − 0.2, *p* = 0.03) (Fig. 2f).

### Association of HDL functions with acute coronary syndrome

**Fig. 3:**
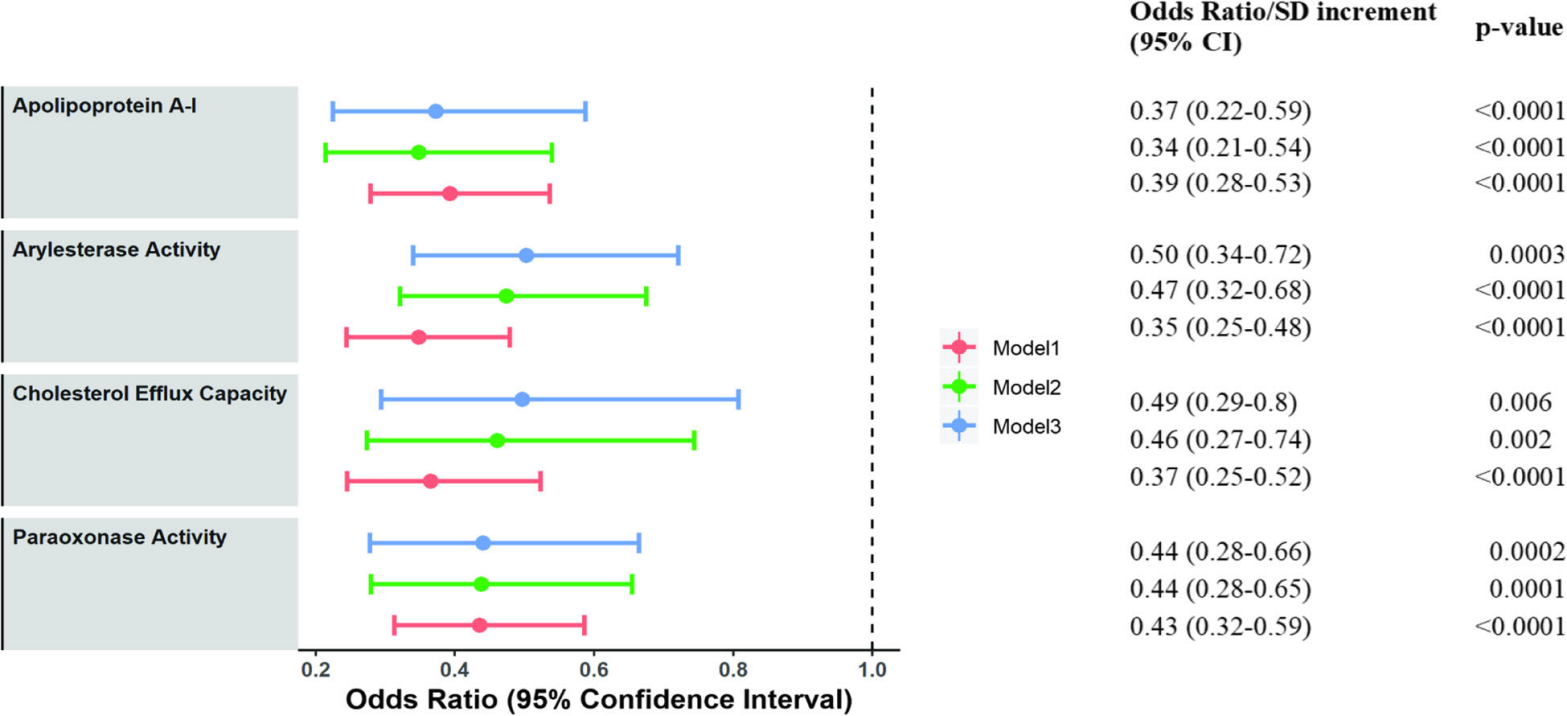
Association of HDL functions with acute coronary syndrome. Odds ratios per 1-SD increase their respective 95% confidence intervals (CIs) and *p* values are shown for acute coronary syndrome. Dots represent odds ratios, and error bars indicate 95% confidence intervals. Logistic regression model 1 indicates unadjusted odds ratio, model 2 adjusted for age, gender, BMI, smoking status, diabetes, hypertension and LDL-C. In model 3, additional adjustment for HDL-C was done. Arylesterase activity represents phenyl acetate hydrolyzing activity and Paraoxonase activity represents paraoxon-hydrolyzing activity of PON1

Logistic regression was performed to analyze the association of apolipoprotein A-I, cholesterol efflux capacity and PON1 activity of HDL with the odds of having ACS (Fig. 3). Apolipoprotein A-I, Cholesterol efflux capacity, PON1 paraoxon-hydrolyzing and phenyl acetate hydrolyzing activities were seen to have a protective effect (Model 1). The effect of apolipoprotein A-I, efflux capacity and antioxidative activity remained significant even after adjustment for age, gender, BMI and other cardiovascular risk factors like smoking, diabetes, hypertension and LDL-C (Model 2) (Fig. 3). Higher cholesterol efflux capacity (odds ratio per 1-SD increase: 0.49; 95% confidence interval: 0.29–0.8; *p* = 0.006), PON1 paraoxon-hydrolyzing activity (odds ratio per 1-SD increase: 0.44; 95% confidence interval: 0.28–0.66; *p* = 0.0002) and phenyl acetate hydrolyzing activity (odds ratio per 1-SD: 0.50; 95% confidence interval: 0.34–0.72; *p* = 0.003) were associated with lower odds of development of ACS even after additional adjustment for HDL-C levels (Model 3).

**Fig. 4:**
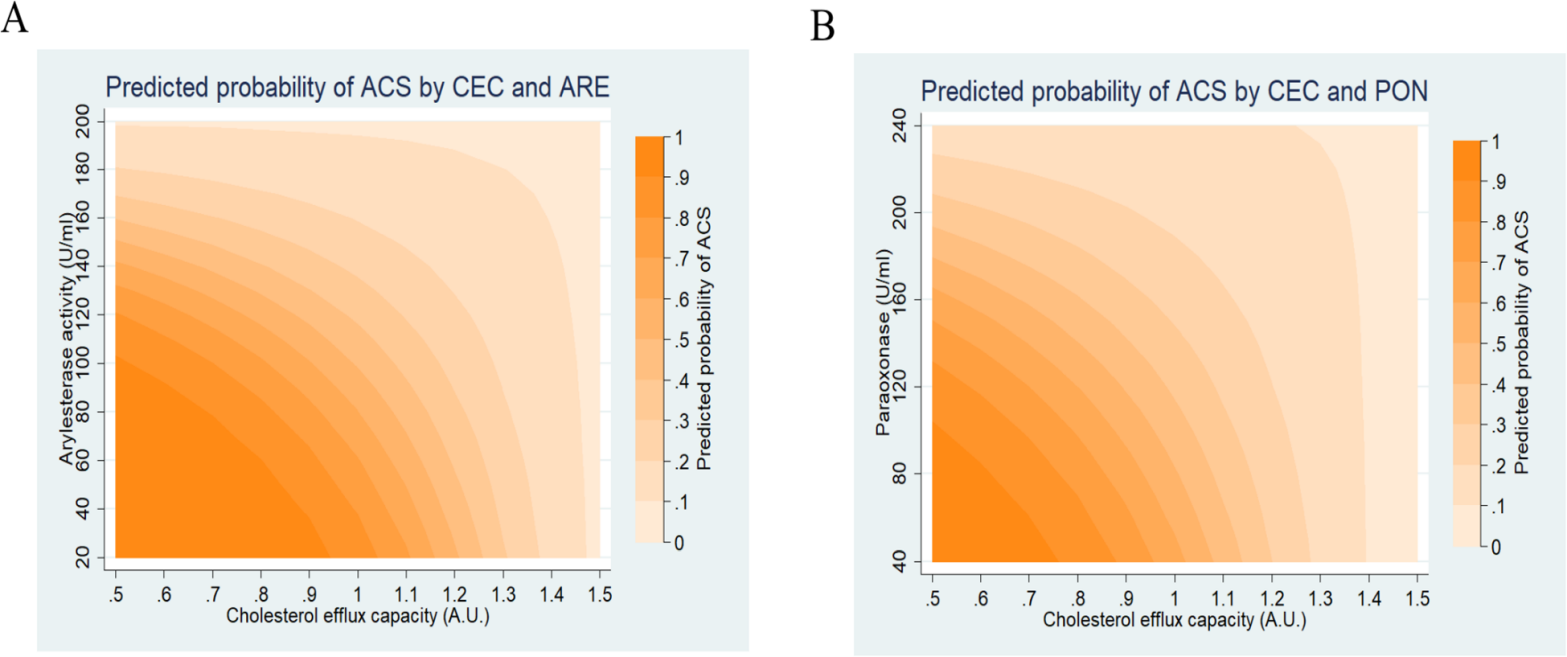
Predicted probability of Acute coronary syndrome. Predicted probability based on interaction between cholesterol efflux capacity (CEC) and (**a**) Arylesterase activity (ARE, phenyl acetate hydrolyzing activity) and (**b**) Paraoxonase activity (PON, paraoxon-hydrolyzing activity) of PON1

We then assessed interactions between HDL functions (cholesterol efflux capacity and arylesterase activity, cholesterol efflux capacity and paraoxonase activity) to predict the probability of ACS. This interaction is meant to represent how the effect of CEC on the predicted probability of ACS differs across levels of phenyl acetate hydrolyzing activity and vice versa (Fig. 4a). Similarly, the effect of CEC on the predicted probability of ACS at different levels of PON (paraoxon-hydrolyzing activity of PON1) was also analyzed as in Fig. 4b. We calculated the predicted probability of acute coronary syndrome for all combinations of cholesterol efflux capacity ranging from 0.5 to 1.5 with an increment of 0.5, and phenyl acetate hydrolyzing activity, ranging from 20 to 210 U/mL (increment 50). Similarly, we also analyzed the interaction of CEC with paraoxon-hydrolyzing activity (ranging from 40 to 240 U/mL with an increment of 10) We observed that an individual with cholesterol efflux capacity (CEC) 1 A.U. and phenyl acetate hydrolyzing activity (ARE) 100 U/mL has a 50% chance of having acute coronary syndrome (*p* < 0.001), while an individual with CEC 1 A.U. and ARE 190 U/mL has a 10% chance of having ACS (*p* = 0.06). We found that the interaction between two HDL functions was significant for lower values of cholesterol efflux capacity and PON1 activity (phenyl acetate hydrolyzing activity and paraoxon-hydrolyzing activity) but was not significant for low cholesterol efflux values and high phenyl acetate hydrolyzing or paraoxon-hydrolyzing values.

## Discussion

In patients presenting with ACS, HDL functions (cholesterol efflux capacity and antioxidative activity) were significantly impaired. Impaired HDL function was found to be associated with higher odds of having ACS even after adjusting for known risk factors including HDL-C levels. At follow-up, there was a significant improvement in the HDL function in these patients despite a marginal(non-significant) decrease in HDL-C levels, though PON1 activities toward paraoxon and phenyl acetate remained lower than in controls. Cholesterol efflux capacity and arylesterase activity were significantly correlated with apolipoprotein A-I levels but not with HDL-C levels. We also found that combination of cholesterol efflux capacity and antioxidative activity strengthened the probability of ACS prediction.

CEC, which represents HDL’s ability to prevent the formation of macrophage foam cells by removing excess cholesterol from macrophages, rather than HDL cholesterol levels, has been shown to have significant negative correlation with atherosclerotic burden [11, 12]. Our results demonstrate that HDL from ACS patients had lower ability to mediate cholesterol efflux from lipid laden macrophages compared to HDL from healthy subjects. These results are consistent with the results obtained in other studies that compared the cholesterol efflux capacity in ACS immediately after the onset of symptoms [13, 14].On the concern of the assay could be that using same amount of apo B depleted serum irrespective of HDL-C levels could affect the measured levels of CEC. However, HDL-C demonstrated no correlation with CEC and even subjects with similar HDL-C had significantly different total macrophage cholesterol efflux capacity [10, 15]. The findings of our study are consistent with the above studies that reported that cholesterol efflux capacity is independent of HDL-C levels. Furthermore, the dal-ACUTE study has shown that treatment with CETP inhibitor, Dalcetrapib increased HDL-C levels by 33.7% while cholesterol efflux capacity was increased only by 9.5% supporting the concept that HDL-C and CEC are delinked [16].

PON1 is an HDL associated anti-atherosclerotic enzyme that prevents the accumulation of lipoperoxides and inhibits the lipid oxidation in low-density lipoproteins (LDL), an observation supported by earlier studies [17–19]. The binding of PON1 to apolipoprotein A-I in HDL modulates its activity [20]. In the present study, we observed lower PON1 activities toward paraoxon and phenyl acetate in ACS group which improved at follow-up but was lower than controls suggesting decreased activity despite optimal therapy.

Our group of acute coronary syndrome patients had a high percentage of smokers and since smoking is one of the risk factors for ACS, we compared the functionality of HDL between smokers and nonsmokers presenting with ACS. We observed a lower HDL efflux capacity and arylesterase activity in smokers presenting with ACS than non-smokers who had ACS. This suggests that oxidative stress induced by smoking to be a major factor for impaired HDL function.

We observed that the inflammatory status improved after therapy as indicated by decrease in hs-CRP by 62%, an effect that could partially be contributed by the statin component of the therapy. In the present study, while a significant improvement and increase was observed in cholesterol efflux capacity and PON1 activity after six months of treatment, the latter continued to be significantly lower than in normal participants even after six months suggesting mechanisms other that acute phase responses.

A study on association of HDL cholesterol efflux capacity with incidence of coronary artery disease using radiolabeled cholesterol reported that the cholesterol efflux capacity was positively associated with apolipoprotein A-I levels and inversely associated with the incidence of coronary disease [21]. Data from our efflux assay performed using BODIPY- cholesterol loaded THP-1 cells, found a similar association between cholesterol efflux capacity and odds of having ACS. Other functional parameters of HDL like PON1 activities toward paraoxon and phenyl acetate were also shown to be protective against development of ACS. CEC of apo B depleted serum and apolipoprotein A-I levels in both control and ACS patients (baseline and follow-up) showed significant correlation and 15% of the variability in cholesterol efflux capacity of HDL could be explained by apolipoprotein A-I levels.

However, we found no correlation between circulating HDL cholesterol levels and cholesterol efflux capacity in control subjects and ACS patients. Furuyama F et al. in their study on effect of cardiac rehabilitation on HDL function have also shown significant correlation of CEC and ARE with apoA-I levels rather than HDL-cholesterol levels [22]. This may be because cholesterol content makes up only about 20% of the HDL particle and even that proportion differs between different HDL particles. This supports the concept that HDL cholesterol level is not an accurate measure of HDL function and marker of CVD risk as seen in recent studies [5]. Therefore, establishing a standardized cell free system for measuring CEC will help clinicians to not only understand the association of cholesterol efflux capacity with cardiovascular disease but also help with identification of high-risk individuals for intervention.

Currently cardiovascular risk stratification is done using parameters like HDL-C and apoA-1 levels. The interaction observed between HDL functions suggests that impairment of both the HDL functions increases the chances of having ACS, while high CEC, ARE and PON values confer protection against ACS. Also, our results indicate that the risk prediction for ACS can be improved by measuring the HDL functional parameters together.

Indians have a high prevalence of low HDL-C levels and cardiovascular disease. However, HDL function has not been studied in them and this is the first study to do so. Currently HDL functional parameters like CEC and PON1 activity are suggested for risk prediction of CVD development very much like how HDL-C and LDL-C levels are used. However, large scale population-based studies are required to establish reference levels for HDL functionality parameters for the assessment of ACS. Whether low HDL function confers additional risk of CVD in Indian population cannot be conjectured since comparative efflux capacity studies with Caucasian population are not available. However, future studies will be useful to evaluate this aspect of CVD risk.

Our study has a few limitations. Two groups were not age and gender matched but we have adjusted for these two factors in our analysis. We evaluated the levels of only one inflammatory marker although other inflammatory and oxidative stress markers are also seen elevated in case of acute coronary syndrome [23] and are responsible for the generating dysfunctional HDL [24]. It has been suggested that acute phase reactions due to systemic inflammation could impair HDL functionality and therefore measuring HDL functionality during acute presentation in ACS patients may not be instructive. In order to explore this aspect, we analyzed the HDL functionality at an additional time point (six months after the presentation of ACS) to understand the contribution of the acute phase to the HDL functionality. We found that the HDL functionality after six months continued to be low as compared to controls despite six months having elapsed and statin therapy. In addition, studies have demonstrated that CEC can serve as a clinical biomarker for cardiovascular risk prediction beyond the traditional validated inflammatory biomarkers like hs-CRP [25]. Thus, the acute phase response is unlikely to be the sole contributor to the differences observed in the HDL functionality. Finally, while the results prove that HDL function is a good predictor for risk of ACS, significant improvement is required in developing a composite measure of HDL function that is adaptable for clinical set up.

The correlation of HDL functions with apolipoprotein A-I along with recent evidence supports the rationale of HDL functions being considered as the predominant therapeutic target for lipid lowering therapy rather than simply its cholesterol mass [26]. Current studies are focused on improving the functions of HDL particles, with one of them, CSL-112, a recombinant apolipoprotein A-I undergoing Phase 3 clinical trials in patients with myocardial infarction to see the effect on major adverse cardiac events (MACE), after Phase 2b trials having shown large increases in efflux capacity with this drug [27, 28]. Our data support the rationale of therapies to improve HDL function to manage the residual risk observed even after optimal decreases in LDL levels in patients with ACS.

## Conclusion

In conclusion, our study supports the concept of HDL function as a superior measure of cardiovascular risk than measuring HDL-cholesterol levels. Standard therapy after ACS improved HDL function partially suggesting remaining residual risk in these patients which maybe future targets of therapy. Our findings also indicate that using a combination of HDL functions [cholesterol efflux capacity and antioxidative activity (as reflected in Paraoxonase 1(PON1) activity)] could improve the predictive accuracy for ACS.

## Data Availability

Not applicable.

## Abbreviations

ACS: Acute coronary syndrome
Apo A-I: Apolipoprotein A-I
Apo B: Apolipoprotein B
ARE: Arylesterase
CEC: Cholesterol efflux capacity
CVD: Cardiovascular disease
HDL-C: High density lipoprotein- cholesterol
hsCRP: High sensitive C reactive protein
LDL-C: Low density lipoprotein – Cholesterol
PON1: Paraoxonase1
TC: Total Cholesterol
TG: Triglyceride
VLDL: Very low density lipoprotein – Cholesterol

## References

[1] Santos-Gallego CG, Badimon JJ, Rosenson RS (2014). Beginning to understand high-density lipoproteins. Endocrinol Metab Clin N Am.

[2] Investigators AIM-HIGH, Boden WE, Probstfield JL (2011). Anderson T, Chaitman BR, Desvignes-Nickens P, et al. niacin in patients with low HDL cholesterol levels receiving intensive statin therapy. N Engl J Med.

[3] Nicholls SJ, Tuzcu EM, Brennan DM, Tardif J-C, Nissen SE (2008). Cholesteryl ester transfer protein inhibition, high-density lipoprotein raising, and progression of coronary atherosclerosis: insights from ILLUSTRATE (investigation of lipid level management using coronary ultrasound to assess reduction of atherosclerosis by CETP inhibition and HDL elevation). Circulation..

[4] Jansen H, Samani NJ, Schunkert H (2014). Mendelian randomization studies in coronary artery disease. Eur Heart J.

[5] Voight BF, Peloso GM, Orho-Melander M, Frikke-Schmidt R, Barbalic M, Jensen MK (2012). Plasma HDL cholesterol and risk of myocardial infarction: a mendelian randomisation study. Lancet..

[6] Nagao M, Nakajima H, Toh R, Hirata K, Ishida T (2018). Cardioprotective effects of high-density lipoprotein beyond its anti-Atherogenic action. J Atheroscler Thromb.

[7] Hafiane A, Genest J (2015). High density lipoproteins: measurement techniques and potential biomarkers of cardiovascular risk. BBA Clin..

[8] Rohatgi A, Khera A, Berry JD, Givens EG, Ayers CR, Wedin KE (2014). HDL cholesterol efflux capacity and incident cardiovascular events. N Engl J Med.

[9] Brites F, Martin M, Guillas I, Kontush A (2017). Antioxidative activity of high-density lipoprotein (HDL): mechanistic insights into potential clinical benefit. BBA Clin.

[10] Sankaranarayanan S, Kellner-Weibel G, de la Llera-Moya M, Phillips MC, Asztalos BF, Bittman R (2011). A sensitive assay for ABCA1-mediated cholesterol efflux using BODIPY-cholesterol. J Lipid Res.

[11] Khera AV, Cuchel M, de la Llera-Moya M, Rodrigues A, Burke MF, Jafri K (2011). Cholesterol efflux capacity, high-density lipoprotein function, and atherosclerosis. N Engl J Med.

[12] Guerin M, Silvain J, Gall J, Darabi M, Berthet M, Frisdal E (2018). Association of Serum Cholesterol Efflux Capacity with Mortality in patients with ST-segment elevation myocardial infarction. J Am Coll Cardiol.

[13] Hafiane A, Jabor B, Ruel I, Ling J, Genest J (2014). High-density lipoprotein mediated cellular cholesterol efflux in acute coronary syndromes. Am J Cardiol.

[14] Annema W, Willemsen HM, de Boer JF, Dikkers A, van der Giet M, Nieuwland W (2016). HDL function is impaired in acute myocardial infarction independent of plasma HDL cholesterol levels. J Clin Lipidol.

[15] de la Llera-Moya M, Drazul-Schrader D, Asztalos BF, Cuchel M, Rader DJ, Rothblat GH (2010). The ability to promote efflux via ABCA1 determines the capacity of serum specimens with similar high-density lipoprotein cholesterol to remove cholesterol from macrophages. Arterioscler Thromb Vasc Biol.

[16] Ray KK, Ditmarsch M, Kallend D, Niesor EJ, Suchankova G, Upmanyu R (2014). The effect of cholesteryl ester transfer protein inhibition on lipids, lipoproteins, and markers of HDL function after an acute coronary syndrome: the dal-ACUTE randomized trial. Eur Heart J.

[17] James RW, Deakin SP (2004). The importance of high-density lipoproteins for paraoxonase-1 secretion, stability, and activity. Free Radic Biol Med.

[18] Viktorinova A, Jurkovicova I, Fabryova L, Kinova S, Koren M, Stecova A (2018). Abnormalities in the relationship of paraoxonase 1 with HDL and apolipoprotein A1 and their possible connection to HDL dysfunctionality in type 2 diabetes. Diabetes Res Clin Pract.

[19] Li C, Chen JW, Ding FH, Shen Y, Liu ZH, Wang F (2019). Relationship of high-density lipoprotein-associated Arylesterase activity to systolic heart failure in patients with and without type 2 diabetes. Sci Rep.

[20] Oda MN, Bielicki JK, Berger T, Forte TM (2001). Cysteine substitutions in apolipoprotein A-I primary structure modulate paraoxonase activity. Biochemistry..

[21] Saleheen D, Scott R, Javad S, Zhao W, Rodrigues A, Picataggi A (2015). Association of HDL cholesterol efflux capacity with incident coronary heart disease events: a prospective case-control study. Lancet Diabetes Endocrinol.

[22] Furuyama F, Koba S, Yokota Y, Tsunoda F, Shoji M, Kobayashi Y (2018). Effects of cardiac rehabilitation on high-density lipoprotein-mediated cholesterol efflux capacity and Paraoxonase-1 activity in patients with acute coronary syndrome. J Atheroscler Thromb.

[23] Al Shahi H, Shimada K, Miyauchi K, Yoshihara T, Sai E, Shiozawa T (2015). Elevated circulating levels of inflammatory markers in patients with acute coronary syndrome. Int J Vasc Med.

[24] Undurti A, Huang Y, Lupica JA, Smith JD, DiDonato JA, Hazen SL (2009). Modification of high density lipoprotein by myeloperoxidase generates a pro-inflammatory particle. J Biol Chem.

[25] Mody P, Joshi PH, Khera A, Ayers CR, Rohatgi A (2016). Beyond coronary calcification, family history, and C-reactive protein: cholesterol efflux capacity and cardiovascular risk prediction. J Am Coll Cardiol.

[26] Danielle D, Rader Daniel J (2006). Emerging therapies targeting high-density lipoprotein metabolism and reverse cholesterol transport. Circulation..

[27] Michael Gibson C, Korjian S, Tricoci P, Daaboul Y, Yee M, Jain P (2016). Safety and tolerability of CSL112, a reconstituted, infusible, plasma-derived Apolipoprotein A-I, after acute myocardial infarction: the AEGIS-I trial (ApoA-I event reducing in ischemic syndromes I). Circulation..

[28] Capodanno D, Mehran R, Gibson CM, Angiolillo DJ (2018). CSL112, a reconstituted, infusible, plasma-derived apolipoprotein A-I: safety and tolerability profiles and implications for management in patients with myocardial infarction. Expert Opin Investig Drugs.

